# Clinicopathologic and endoscopic characteristics of ten patients with gastric hamartomatous inverted polyp: a single center case series

**DOI:** 10.1186/s12876-024-03233-8

**Published:** 2024-04-22

**Authors:** Ningning Dong, Fandong Meng, Bing Yue, Junzhen Hou

**Affiliations:** 1 Department of Gastroenterology, Beijing Friendship Hospital, Capital Medical University, State Key Laboratory for Digestive Health, National Clinical Research Center for Digestive Diseases, Beijing Digestive Disease Center, Beijing Key Laboratory for Precancerous Lesion of Digestive Diseases, Beijing, 100050 China; 2 Department of Pathology, Beijing Friendship Hospital, Capital Medical University, Beijing, 100050 China; 3 Department of Gastroenterology, Shijingshan Teaching Hospital of Capital Medical University, Beijing Shijingshan Hospital, 24 Shi-Jing-Shan Road Shi-Jing-Shan District, Beijing, 100040 China

**Keywords:** Gastric hamartomatous inverted polyp, Clinicopathologic characteristic, Gastritis, Gastric atrophy

## Abstract

**Background:**

Gastric hamartomatous inverted polyps (GHIPs) are not well characterized and remain diagnostically challenging due to rarity. Therefore, this study aims to investigate the clinicopathologic and endoscopic characteristics of patients with GHIP.

**Methods:**

We retrospectively reviewed clinicopathologic and endoscopic features of ten patients with GHIP who were admitted to Beijing Friendship Hospital from March 2013 to July 2022. All patients were treated successfully by endoscopic resection.

**Results:**

GHIPs were usually asymptomatic and found incidentally during gastroscopic examination. They may be sessile or pedunculated, with diffuse or local surface redness or erosion. On endoscopic ultrasonography, the sessile submucosal tumor-type GHIP demonstrated a heterogeneous lesion with cystic areas in the third layer of the gastric wall. Histologically, GHIPs were characterized by a submucosal inverted proliferation of cystically dilated hyperplastic gastric glands accompanied by a branching proliferation of smooth muscle bundles. Inflammatory cells infiltration was observed in the stroma, whereas only one patient was complicated with glandular low-grade dysplasia. Assessment of the surrounding mucosa demonstrated that six patients (60%) had atrophic gastritis or *Helicobacter pylori*–associated gastritis, and four patients (40%) had non-specific gastritis. Endoscopic resection was safe and effective.

**Conclusions:**

GHIPs often arise from the background of abnormal mucosa, such as atrophic or *H.pylori*-associated gastritis. We make the hypothesis that acquired inflammation might lead to the development of GHIPs. We recommend to make a full assessment of the background mucosa and *H. pylori* infection status for evaluation of underlying gastric mucosal abnormalities, which may be the preneoplastic condition of the stomach.

## Background

Gastric hamartomatous inverted polyps (GHIPs), characterized by the downward growth of hyperplastic mucosal component into the submucosal layer [1], account for fewer than 1% of all gastric polyps [2]. They have also been called gastric inverted hyperplastic polyps(GIHPs) [3–6], because of the similarity to their colonic counterpart [7]. Collectively, lesions exhibiting inverted growth are referred to as “gastric inverted polyps (GIPs)” [2]. Kim et al. [8] divided gastric inverted polyps (GIPs) into three subtypes based on their communication with the mucosal surface, smooth muscle boundary, and tissue organization. Type 1 has a central mucosal communicating structure and a recognizable smooth muscle boundary, and has a typical round vase shape when viewed under low magnification. Half of type 1 may be accompanied by simultaneous cancer transformation. Type 2 is similar to type 1 but with no central communicating structure. Type 3 is characterized by distorted lobular tissue organization composed of cystic or hyperplastic glands and smooth muscle, without a mucosal communicating structure or smooth muscle boundary.

Because of their rarity, GHIPs are not well characterized and remain diagnostically challenging based on endoscopic findings [1]. Moreover, the pathogenesis and precancerous potential of GHIPs are still uncertain, meanwhile their association with various forms of gastritis has not been well documented in the literature. Herein we retrospectively reviewed clinical, endoscopic, and histological data of ten patients with GHIPs, all of which were resected successfully by endoscopy.

## Methods

This retrospective study was approved by the Ethic Committee of Beijing Friendship Hospital, Capital Medical University (Beijing, China). Written informed consent was obtained from all patients. A total of ten patients with GHIP were included in the study from March 2013 to July 2022. None of the patients had prior gastric surgery or a family history of gastric cancer or gastrointestinal polyposis syndromes. The demographics, clinical manifestations, endoscopic and histopathological features were obtained from the patients’ medical records. Endoscopic mucosal resection (EMR) or endoscopic submucosal dissection (ESD) was performed for GHIPs. *Helicobacter pylori* (*H. pylori*) infection status was assessed by ^13^C-urea breath test (UBT) (Shenzhen China National Nuclear Corporation Heidewe Biotechnology, China), past history of prior successful *H. pylori* eradication therapy, microscopic observations of biopsied/resected specimens, serum *H. pylori* antibody test, endoscopic manifestations [9], or a combination of these methods. ^13^C-UBT was performed in the morning after a at least of 6 h fasting, with no close (within the past 4 weeks) or concomitant medical history of proton pump inhibitors, antibiotics and bismuth, with a dosage of Urea of 75 mg, and the cut-off value to distinguish whether the ^13^C-UBT is positive or negative was defined as 4‰. Based on the results of these tests, we divided the patients into two groups according to the *H. pylori* infection status: Hp group (consisting of patients with current or past *H. pylori* infection) and uninfected group (consisting of *H. pylori*-uninfected patients).

The histopathological findings were analyzed by hematoxylin and eosin (HE) staining and immunohistochemical staining (for Mucin 6, Mucin-5AC, Mucin 2, Pepsinogen I and Desmin), including the glandular components (foveolar, fundic, cardiac/pyloric/mucous-neck, and intestinal type), the presence of epithelial dysplasia or not, and the characteristics of stroma, muscularis mucosae and background gastric mucosa. The exact sample size was a total of ten lesions from ten patients. All the submitted specimens were fixed with 10% neutral formaldehyde solution, followed by routine dehydration, paraffin embedding, tissue sectioning at a thickness of 4 µm and HE staining. An En-Vision two-step method was used for immunohistochemical labelling. The pepsinogen-I antibody was purchased from Abcam Company in the United States. Other antibodies (including Mucin 6, Mucin-5AC, Mucin 2 and Desmin) were purchased from Fuzhou Maixin Medical Technology Co., Ltd. Negative and positive controls were established for the above markers.

Subsequently, we investigated the atrophy status of the gastric mucosa surrounding/overlying each GHIP endoscopically (according to “Kimura and Takemoto's endoscopic-atrophic border scale [10, 11]”) and pathologically. In biopsy/resected specimens, mucosa with glandular atrophy or metaplasia (including focal or extensive intestinal/pseudo-pyloric metaplasia) was determined to be atrophic gastritis.

We compared the main clinical and endoscopic characteristics of GHIPs between patients with and without *H. pylori* infection. The statistical analysis was performed using R language Statistical Software (R 4.3.2). Fisher's precision probability test was used to compare categorical variables, and the independent-sample t test for quantitative variables.

## Results

### Clinical and endoscopic findings

**Fig. 1:**
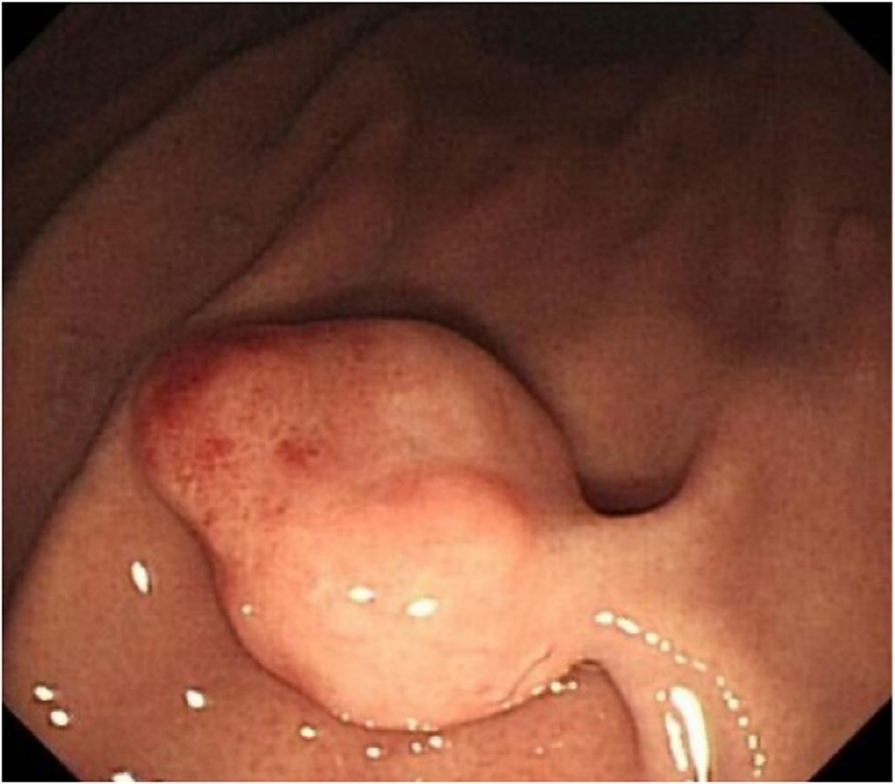
Image of endoscopy of a pedunculated polyp-type GHIP in the gastric body without mucosal diffuse redness or atrophy

**Fig. 2:**
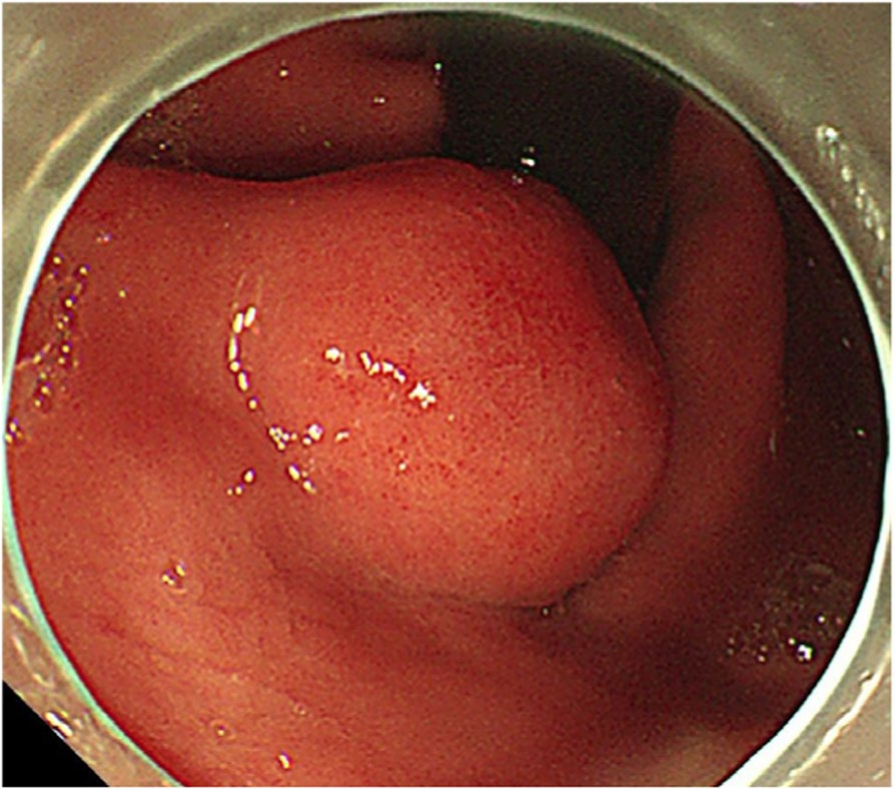
Image of endoscopy of a sessile submucosal tumor (SMT)-type GHIP in the gastric cardia

**Fig. 3:**
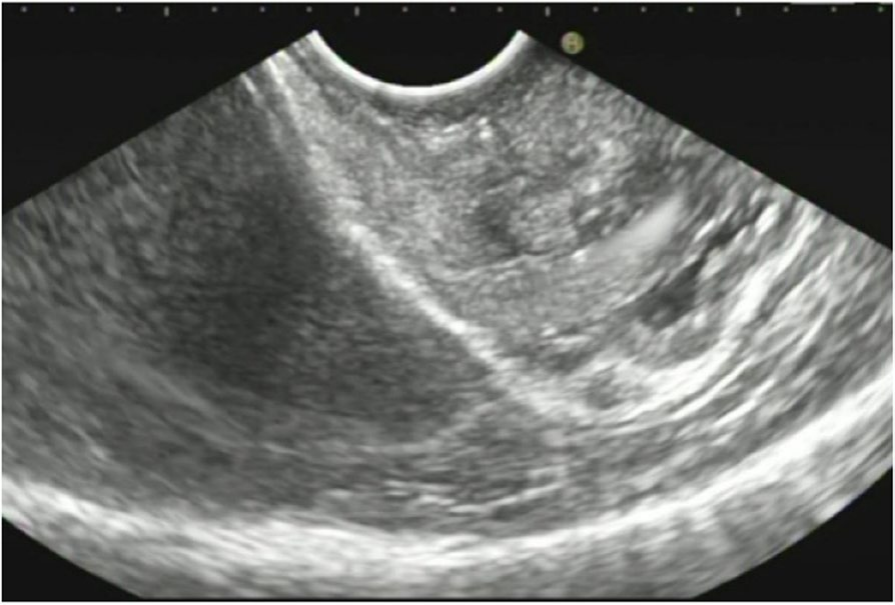
Endoscopic ultrasound (radial scan, 10 MHz) revealed the GHIP as a heterogeneous tumor with multiple small hypoechoic or anechoic areas in the third layer of the gastric wall

**Fig. 4:**
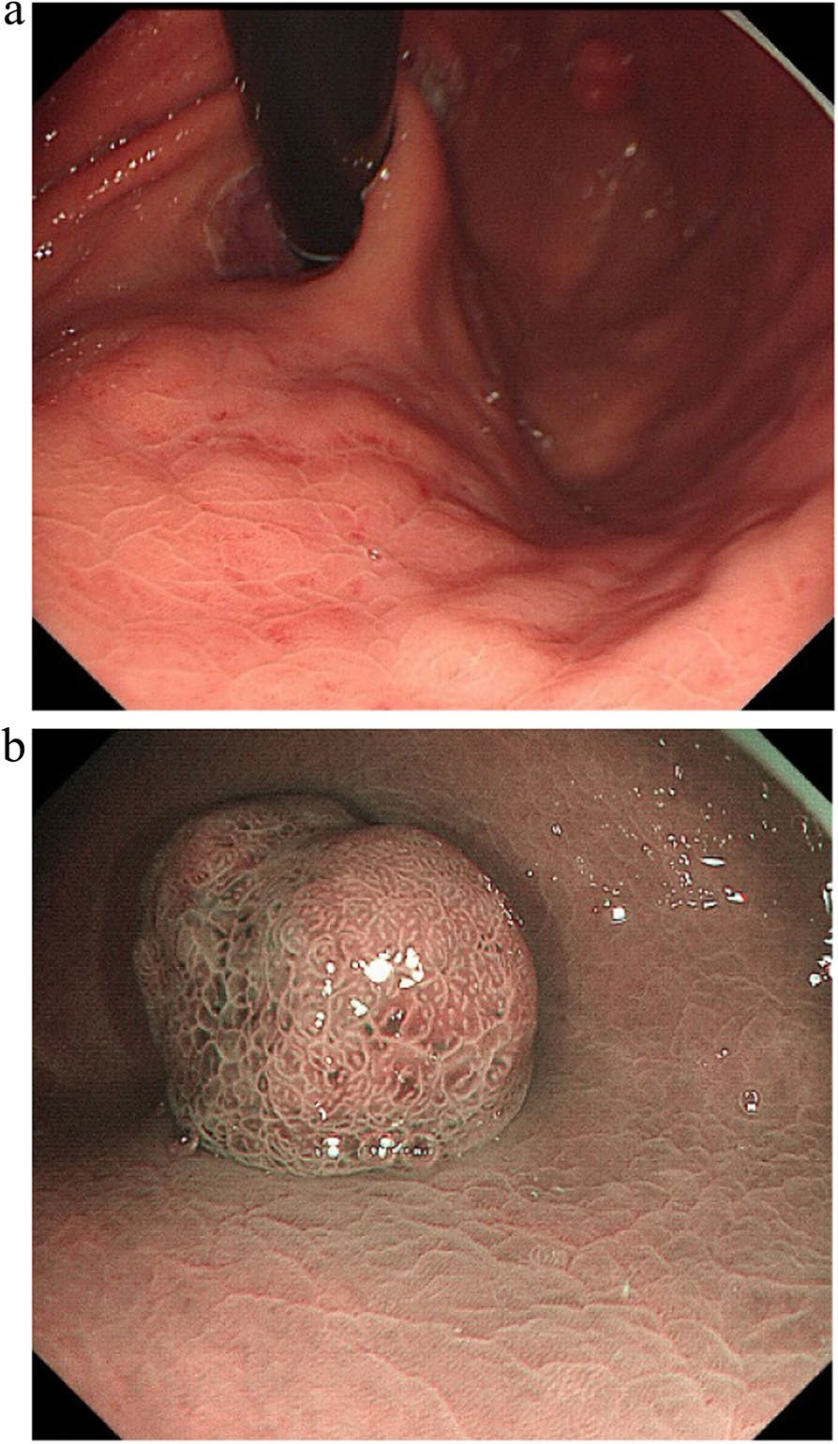
Images of endoscopy of a GHIP in the gastric fundus with mucosal diffuse redness, which indicated *H. pylori* currently infection (**a** white light image. **b** narrow-band image)

A summary of the clinical and endoscopic findings in patients with GHIP was shown in Table 1. GHIPs typically presented in late adulthood (median age of diagnosis, 59.5 years old; range, 42–79 years old), with a modest male predominance (7/10, 70%). They located in the proximal stomach (four in the middle-upper body, four in the fornix and two in the cardia), with the maximum diameter ranging from 6 to 20 mm (median size, 13.5 mm). Most patients were asymptomatic and diagnosed incidentally during endoscopic examination, however, a minority (3/10, 30%) presented with non-specific symptoms, including heartburn, acid regurgitation and epigastric distension. Endoscopically, GHIPs were solitary, and could be classified into pedunculated polyp-type (Fig. 1) (7/10, 70%), which were all completely resected by EMR, and sessile submucosal tumor (SMT)-type (Fig. 2) (3/10, 30%), which were all completely resected by ESD. All of the ten GHIPs exhibited diffuse (3/10, 30%) or local (7/10, 70%) surface redness or erosion. On endoscopic ultrasonography (EUS), all of the three sessile SMT-type GHIPs demonstrated a heterogeneous lesion with anechoic cystic areas in the third layer of the gastric wall (Fig. 3). *H. pylori*-infection of the gastric mucosa was confirmed in four cases (4/10, 40%), including three patients with current infection (Fig. 4a and b) and one patient with past infection. All patients were discharged without any significant complications after the endoscopic resection.

**Table 1:** Clinical and endoscopic findings in patients with GHIPs

No	Gender	Age	Location	Size (mm)	Endoscopic appearance	Redness/erosion	Symptom	*H.pylori-* infection status	Procedure
1	Male	78	cardia	20*12	sessile	+	heartburn, acid regurgitation	Cur	ESD
2	Female	48	fornix	15*12	pedunculated	+	None	Cur	EMR
3	Male	79	cardia	6*6	pedunculated	+	heartburn, acid regurgitation	Pre	EMR
4	Male	70	body	12*10	pedunculated	+	None	Cur	EMR
5	Male	45	body	15*10	pedunculated	+	None	N	EMR
6	Female	49	fornix	10*10	sessile	+	None	N	ESD
7	Male	59	body	10*10	pedunculated	+	None	N	EMR
8	Male	60	fornix	20*20	sessile	+	epigastric distension	N	ESD
9	Male	42	body	20*15	pedunculated	+	None	N	EMR
10	Female	74	fornix	10*10	pedunculated	+	None	N	EMR

*H.pylori-* infection status (*Cur* currently infected, *Pre* previously infected, *N* uninfected with *H. pylori*), *EMR* endoscopic mucosal resection, *ESD* endoscopic submucosal dissection

In order to investigate the correlation to *Helicobacter pylori* infection status, we compared the age, gender, morphology, maximum diameter and location of GHIPs between patients with and without *H. pylori* infection. The differences were not statistically significant (*P* > 0.05), as shown in Table 2.

**Table 2:** Comparison of the age, gender, morphology, maximum diameter and location of GHIPs between patients with and without *H. pylori* infection

	HP Group (*n* = 4 cases)	Uninfected Group (*n* = 6 cases)	*P* value
Age, years, Mean (SE)	68.75 (7.20)	54.83 (4.85)	0.133
Gender, n (%)			> 0.999
Male	3 (75)	4 (66.67)	
Female	1 (25)	2 (33.33)	
Maximum diameter, mm, Mean (SE)	13.25 (2.93)	14.17 (2.01)	0.795
Location, n (%)			0.371
Cardia	2 (50)	0 (0)	
Body	1 (25)	4 (66.67)	
fornix	1 (25)	2 (33.33)	
Endoscopic appearance, n (%)			> 0.999
Sessile	1 (25)	2 (33.33)	
pedunculated	3 (75)	4 (66.67)	
Redness/erosion positive, n (%)	4 (100)	6 (100)	

*SE* standard error

### Histopathological findings

**Fig. 5:**
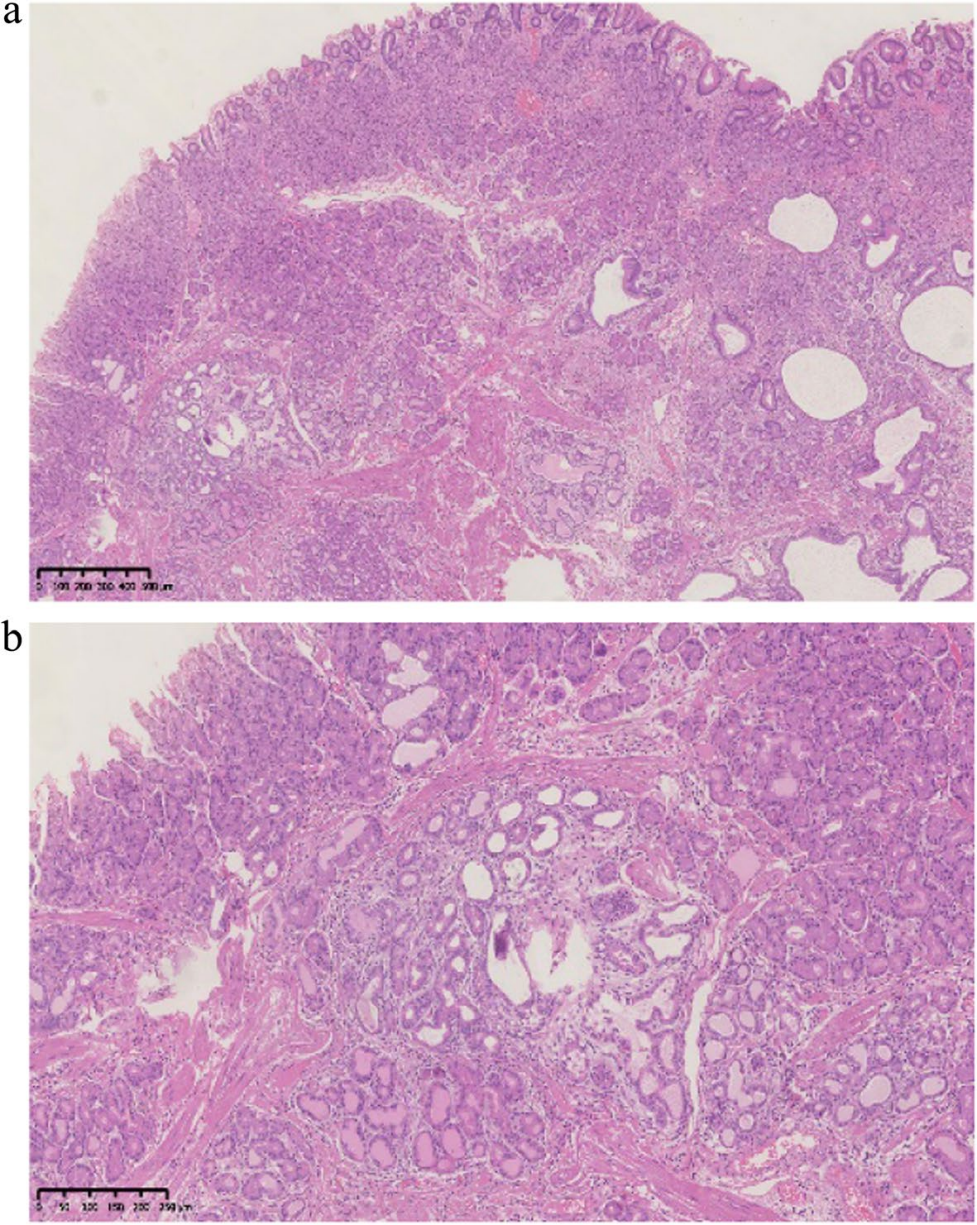
**a** Low-power view of HE staining illustrated the inverted growth lesion, which consisted of dilated glands in various sizes and shapes in the submucosa. **b** Medium-power magnification demonstrated foveolar and mucous-neck glands without cytological atypia and partial cystic dilation

**Fig. 6:**
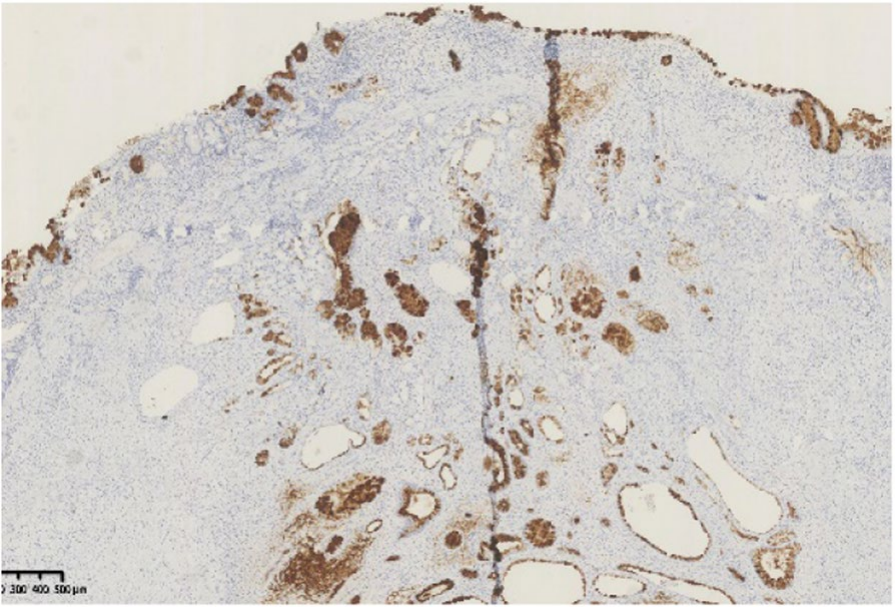
The foveolar epithelium of the overlying mucosa and foveolar type glands in the submucosal lesion were positive for the mucin-5AC immunohistochemical stain

**Fig. 7:**
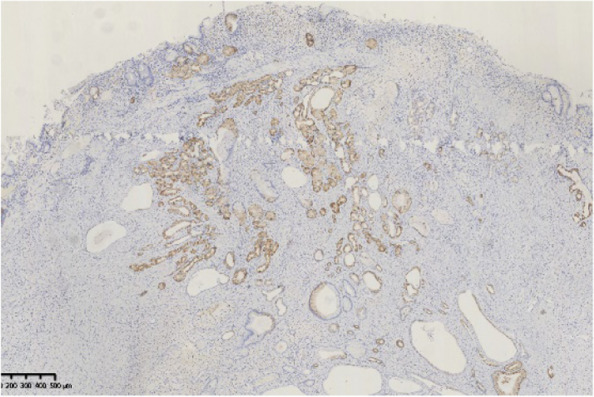
Immunohistochemical stain for mucin 6 showed positive glands in both the overlying mucosa and the submucosal lesion

**Fig. 8:**
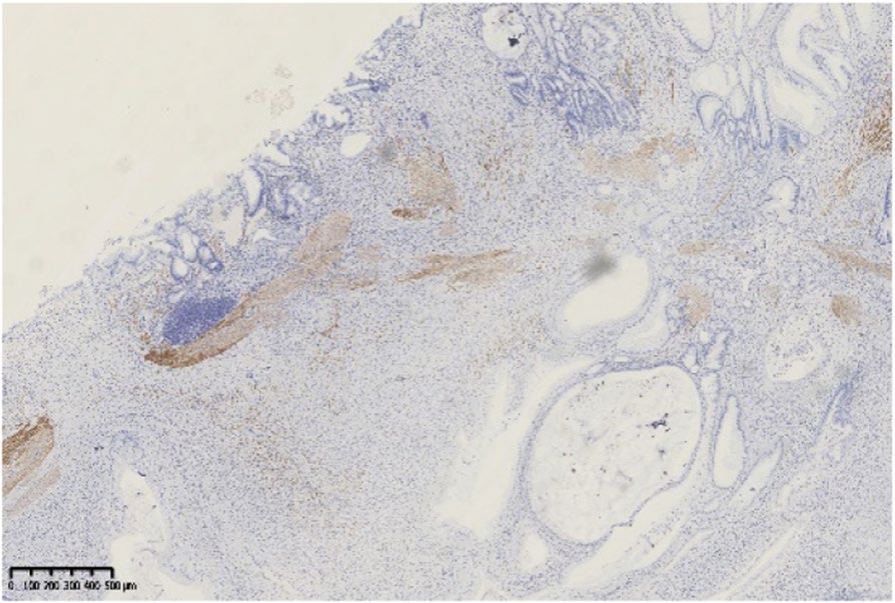
Immunohistochemical stain for Desmin showed the submucosal glands or cystic elements were connected with the overlying gastric mucosa through defects of the muscularis mucosa

Histopathological findings in the ten GHIPs were summarized in Table 3. The histopathological examination of the ten GHIPs revealed well-circumscribed and lobulated submucosal proliferation of cystically dilated hyperplastic glands and smooth muscle bundles, partly including fibroblast cells and calcification (Fig. 5a and b). Within the GHIPs, the glandular structures mainly consisted of foveolar type (Fig. 6), cardiac/pyloric/mucous-neck type epithelium (Fig. 7), meanwhile a small quantity of fundic type or intestinal metaplasia cells were found in four cases. The continuity between the submucosal glands or cystic elements and the overlying gastric mucosa through a defect of the muscularis mucosa was observed in six GHIPs (6/10, 60%) (Fig. 8). Inflammatory cell infiltration was observed in the submucosal stroma within all the ten GHIPs. Only one GHIP (1/10, 10%) complicated with submucosal glandular low-grade dysplasia, but none was accompanied by adenocarcinoma. Assessment of the surrounding mucosa demonstrated that six patients (60%) had *H. pylori*–associated gastritis or atrophic gastritis with intestinal metaplasia (one of them was diagnosed as autoimmune gastritis), and four patients (40%) had non-specific gastritis.

**Table 3:** Histologic findings in the ten GHIPs

No	Stromal inflammati-on	Foveol-ar type	Cardiac/pyloric/mucous-neck type	Intestinal type	Fundic type	Contin-uity	Dysplasia/carcinoma in GHIP	Surrounding gastric mucosa (K-M)
1	+	+	+	-	-	+	-	HAG + AG + IM (O1)
2	+	+	+	-	-	+	-	HAG
3	+	+	+	+	+	+	LGIN	HAG + AG + IM (O2)
4	+	+	+	-	-	+	-	HAG
5	+	+	+	-	-	-	-	NSG
6	+	+	+	+	+	+	-	AG + IM (O1)
7	+	+	+	-	-	-	-	NSG
8	+	+	+	-	+	+	-	AIG (O3)
9	+	+	+	-	-	-	-	NSG
10	+	+	+	-	+	-	-	NSG

*LGIN* low grade intraepithelial neoplasia, *AIG* autoimmune gastritis, *AG* atrophic gastritis, *IM* intestinal metaplasia, *HAG H.pylori*–associated gastritis, *NSG* non-specific gastritis, *K-M* Kimura and Takemoto's endoscopic-atrophic border scale

## Discussion

In the present study, we reported the clinicopathologic and endoscopic features of ten patients with GHIPs (the second largest case study of GIPs up to now). Furthermore, a literature review was conducted on previously reported cases of GIPs in PubMed using various keywords such as 'gastric inverted polyp', 'gastric inverted hyperplastic polyp', or 'gastric hamartomatous inverted polyp' between January 1978 and May 2023. To our knowledge, only 45 cases of GHIPs have been reported in English [1, 3, 5–8, 12–31]. Table 4 summarizes the clinicopathological and endoscopic characteristics of these patients. Our review of the previously reported patients, as well as the ten present patients (totally 55 patients and 56 lesions), revealed a slight male predominance (33 males and 22 females) and a median age at diagnosis of 58 years (range: 23–81 years). Most of the patients were asymptomatic and found incidentally. However, some patients (13/55, 23.6%) presented with non-specific symptoms, including epigastric pain/discomfort, dyspepsia or heartburn and acid regurgitation. Furthermore, few patients presented with anemia secondary to chronic hemorrhage [3, 5, 14] and gastrointestinal obstruction [28] due to the lesion. The vast majority of GHIPs (54/56, 96.4%) located in the proximal stomach and the most common location was in the body (40/56, 71.4%), followed by the fundus (11/56, 19.6%), cardia (3/56, 5.4%) and antrum (2/56, 3.6%). The median diameter was 17 mm (range, 5–45 mm). The vast majority of the patients (54/55, 98.2%) had only one GHIP in the stomach, except for one patient [5] (1/55, 1.8%) who had 2 GHIPs.

**Table 4:** Clinicopathological and endoscopic characteristics of the previously reported 45 patients with GHIPs

	Sessile SMT-type (*n* = 35 patients, 36 lesions)	pedunculated Polyp-type (*n* = 10 patients, 10 lesions)
Median Age (years)	58(23–81)	60(34–75)
Gender (Male/Female)	21/14	5/5
Median maximum diameter (range, mm)	18 (5–40)	21 (10–45)
Location	Body:29; fundus:5; Antrum:1; cardia:1	Body: 7; fundus: 2; Antrum: 1
Surrounding gastric mucosa^a^	Intestinal metaplasia: 10; atrophic gastritis: 9; *H.pylori*-associated gastritis: 4; non-specific gastritis: 6; not available:16	Intestinal metaplasia: 2; atrophic gastritis: 4; *H.pylori*-associated gastritis: 2; non-specific gastritis: 1; not available: 3
Coexisting carcinoma in GIPs	5	1
Separate carcinoma	8	0
Treatment	ESD:15; EMR:1; gastrectomy:6; wedge resection:4; Not available:10	ESD:1; EMR: 2; polypectomy:3; gastrectomy: 2; Not available: 2

^a^Patients with multiple histologic patterns of gastritis were included in multiple categories

The endoscopic manifestations of GHIPs were diverse. Aoki et al. [17] classified the appearances of GHIPs into sessile SMT-type and pedunculated polyp-type on endoscopy. According to this classification, sessile SMT-type was more frequently noted in GHIPs (38/56, 67.9%). Typically, the surface of GHIPs was covered with almost intact gastric mucosa, and an erosive redness or depression was frequently noted, which would indicate the relationship between GHIPs and mucosal inflammation, meanwhile a central orifice or dell with or without milky mucus outflow was occasionally observed, which would indicate the communication between submucosal lesion and gastric lumen. On EUS, the majority demonstrated a heterogeneous lesion with multiple anechoic cystic areas in the third layer of the gastric wall (23/29, 79.3%), however, a minority demonstrated a hypoechoic lesion (6/29, 20.7%). It is difficult to distinguish a GHIP with or without adenocarcinoma based on the EUS manifestation**s.**

Furthermore, the assessment of the surrounding mucosa in the 55 patients revealed the presence of atrophic gastritis/intestinal metaplasia in 21 patients (among them 1 patient was diagnosed as autoimmune gastritis), *H.pylori*-associated gastritis in 10 patients, non-specific chronic gastritis in 11 patients, and data not available in 19 patients. The continuity between the submucosal glands or cystic elements and the overlying gastric mucosa through a defect of the muscularis mucosa or direct communication with the gastric mucosa was observed in 24 GIPs, suggesting that the polyp may have been formed by the heterotopic inverted downgrowth of mucosal glands into the submucosa. In addition, infiltration of chronic inflammatory cells was observed in the submucosal stroma within all GHIPs. According to all these findings, although the pathogenesis of GIPs is unknown, the heterotopic inverted downgrowth of mucosal components in GIPs is thought to develop as a result of infiltration of the mucosa through the muscularis, mucosal crevices or defects caused by repeated erosion due to various types of chronic gastritis [32]. Smooth muscle proliferated bundles would be induced by the regenerative process of both the mucosa and muscularis mucosae caused by repeated erosion [7], supporting the view of GIPs as regenerative lesions as well.

According to the classification of Kim et al. [8], the present study consisted of type 2 and type 3 GHIPs, and within the lesions no carcinomatous component was observed. However, although the exact association between gastric adenocarcinoma and GHIP is still controversial, a few studies reported GHIP coexisted with adenocarcinoma within the lesion [8, 24, 27, 33], or outside the lesion presented as synchronous or metachronous gastric adenocarcinoma [5–8, 25]. The surrounding mucosa was assessed in 9 out of the 14 patients accompanied by adenocarcinoma, revealing *H. pylori*–associated gastritis or atrophic gastritis with intestinal metaplasia in eight patients (8/9, 88.9%), and non-specific chronic gastritis in only one patient. All the six GHIPs coexisted with adenocarcinoma within the lesion were classified into type 1 [8], characterized by a central mucosal communicating structure, which may be the reason for neoplasia because it allowed a continuous exposure to luminal carcinogen and mechanical stress [8]. In four out of the six patients, a hyperplasia–dysplasia–carcinoma sequence was noted within the lesion [8, 24, 33], which indicated that adenocarcinoma might originate from a benign GHIP. Moreover, Ohtsu et al. [14] reported three patients with GHIP whose gene analysis demonstrated no significant mutation. It seems that GHIPs may not be premalignant lesions, but the gastric mucosa with *H. pylori*–associated gastritis or atrophic gastritis with intestinal metaplasia in or outside the polyp is more likely to harbor an adenocarcinoma. Therefore, one important implication of GHIPs appears to be a marker for an abnormal gastric mucosal background that is associated with the development of gastric cancer.

Moreover, as is known to all, gastrin plays a key role in gastric physiology, including various cellular processes such as proliferation, differentiation, angiogenesis, and apoptosis [34–36]. Gastric mucosal inflammation and hypergastrinemia, especially due to atrophic gastritis in oxyntic mucosa and *H.pylori* infection may play a major role in the development and neoplasia of GIPs as a result of repair, regeneration and proliferation. Additional clinicopathological studies are needed to further clarify the pathogenesis of GIPs and the association between the development of GHIPs and precancerous potential with various forms of gastritis.

In terms of treatment, endoscopic diagnosis of a GIP and neoplastic potential within a GIP may be difficult, and biopsy often faces incomplete pathological sampling of the remaining masses, therefore, complete resection may be required for subsequent pathological examination. GHIPs need to be differentiated from ectopic pancreas, gastritis cystica profunda (GCP), gastrointestinal stromal tumor (GIST) and neuroendocrine tumor (NET) [1, 17, 31], mainly through pathological characteristics. The key points of differentiation are as follows: (1) Ectopic pancreas: Microscopically pancreatic acini and ducts can help to distinguish from GHIP, (2) GCPs usually locate at the anastomotic site of the gastrointestinal tract, and the submucosal glands are composed of simple glands without obvious proliferative changes. (3) GIST and NET, as common submucosal tumors in the stomach, can be distinguished from GHIP through immune phenotypes (including immunohistochemical staining for CD34、CD117、DOG1 and Chromogranin A).The polyp-type GHIPs can be resected endoscopically by EMR, but for SMT-type especially larger than 20 mm in diameter, ESD is practical for en bloc resection currently [1, 3, 6, 13, 14, 16, 18, 19, 21, 24–26, 28, 29, 37], which is consistent with our present 10 cases. However, it has also been reported that laparoscopic resection may be suitable for SMT-type GHIPs with a diameter more than 20 mm [17, 23, 27, 33].

## Conclusion

In summary, we present a comprehensive clinicopathologic analysis of 10 GHIPs with a literature review. GHIPs often arise from the background of abnormal mucosa, such as atrophic or *H.pylori*-associated gastritis. We make the hypothesis that acquired inflammation might lead to the development of GHIPs, and the gastric mucosa with H. pylori–associated gastritis or atrophic gastritis with intestinal metaplasia, whether inside or outside the polyp, is more likely to harbor an adenocarcinoma. We recommend to make a full assessment of the background mucosa and *H. pylori* infection status, so as to evaluate the underlying gastric mucosal abnormalities, which may be the preneoplastic condition of the stomach. Nonetheless, considering the limited number of cases and the nature of retrospective analysis in this study, further studies are needed.

## Data Availability

The authors confirm that the data supporting the findings of this study are available within the article.

## Abbreviations

GHIP: Gastric hamartomatous inverted polyp
GIHP: Gastric inverted hyperplastic polyp
GIP: Gastric inverted polyp
EMR: Endoscopic mucosal resection
ESD: Endoscopic submucosal dissection
H. pylori: Helicobacter pylori
UBT: Urea breath test
HE: Hematoxylin and eosin
SMT: Submucosal tumor
EUS: Endoscopic ultrasonography
LGIN: Low grade intraepithelial neoplasia
AIG: Autoimmune gastritis
AG: Atrophic gastritis
IM: Intestinal metaplasia
HAG: H.pylori–associated gastritis
NSG: Non-specific gastritis
GCP: Gastritis cystica profunda
GIST: Gastrointestinal stromal tumor
NET: Neuroendocrine tumor
